# Aortic erosion occurring in over 5 years after Amplatzer septal Occluder implantation for secundum atrial septal defect: a case report

**DOI:** 10.1186/s13019-019-0982-z

**Published:** 2019-09-06

**Authors:** Yasuko Onakatomi, Toshihide Asou, Yuko Takeda, Hideaki Ueda, Motohiko Goda, Munekata Masuda

**Affiliations:** 1 Department of Cardiovascular Surgery, Kanagawa Children’s Medical Center, Yokohama, Japan; 2 Department of Cardiology, Kanagawa Children’s Medical Center, Yokohama, Japan; 3 Department of Cardiovascular Surgery, Yokohama City University, Fukuura 3-9, Kanazawa-ku, Yokohama, Kanagawa 236-0008 Japan

**Keywords:** Atrial septal defect, Erosion, Amplazter septal occluder, Cardiac tamponade, Aortic rim

## Abstract

**Background:**

Aortic erosion is a serious complication that usually occurs shortly after Amplazter Septal Occluder (ASO) implantation for atrial septal defect (ASD).

**Case presentation:**

A seven-year-old girl was diagnosed with secundum ASD without symptoms. Transesophageal echocardiography (TEE) showed a defect of 20 mm in diameter in the fossa ovalis without aortic rim. An ASO device of 24 mm in diameter was selected and electively implanted. The “A-shape” of the device was confirmed by intraoperative TEE, a landmark finding indicating the proper implantation of ASO in patients without aortic rim. After an uneventful postoperative course of 5 years and 10 months, she was transferred to our unit due to cardiogenic shock. Her echocardiogram in emergency room showed pericardial effusion with collapsed right ventricle. Given her history of ASO and the observation of the sequentially increasing pericardial effusion, we diagnosed her with acute cardiac tamponade due to aortic erosion. Emergency pericardiotomy was then performed to improve the hemodynamic condition. Fresh clots were found, so we immediately prepared the cardiopulmonary bypass circuit and explored the damage to the aorta, in which the clots had accumulated. Bleeding suddenly started when the clots were removed. We then inserted the cannulae for perfusion and venous drainage. The clots were removed, and tears were found in both the lateral side of the ascending aorta and the right atrial wall. Intraoperative TEE showed that an edge of the ASO device was directly touching the aortic wall and the Doppler color-flow imaging showed blood flow through this lesion. The erosive lacerations of both the ascending aorta and right atrium were detected from the inside after achieving cardioplegic cardiac arrest. The ascending aorta was obliquely incised, and the laceration was closed from inside the aortic root. The postoperative course was uneventful. She has been doing well for 5 years since the surgery.

**Conclusions:**

We experienced and successfully treated a rare case of acute cardiac tamponade caused by aortic erosion 5 years and 10 months after ASO implantation.

## Background

The Amplazter Septal Occluder (ASO; St. Jude Medical, Plymouth, MN, USA) has been approved for the treatment of secundum atrial septal defect (ASD) since 2005 in Japan. It has been a great boon to a certain number of patients with ASD because it is less invasive and safer than open heart surgery [1]. However, it is associated with a serious complication in aortic erosion, which has been reported to occur in the acute phase, usually within 24 h after ASO implantation [1].

We herein report a rare case in whom aortic erosion occurred in the late phase after ASO implantation.

## Case presentation

**Fig. 1:**
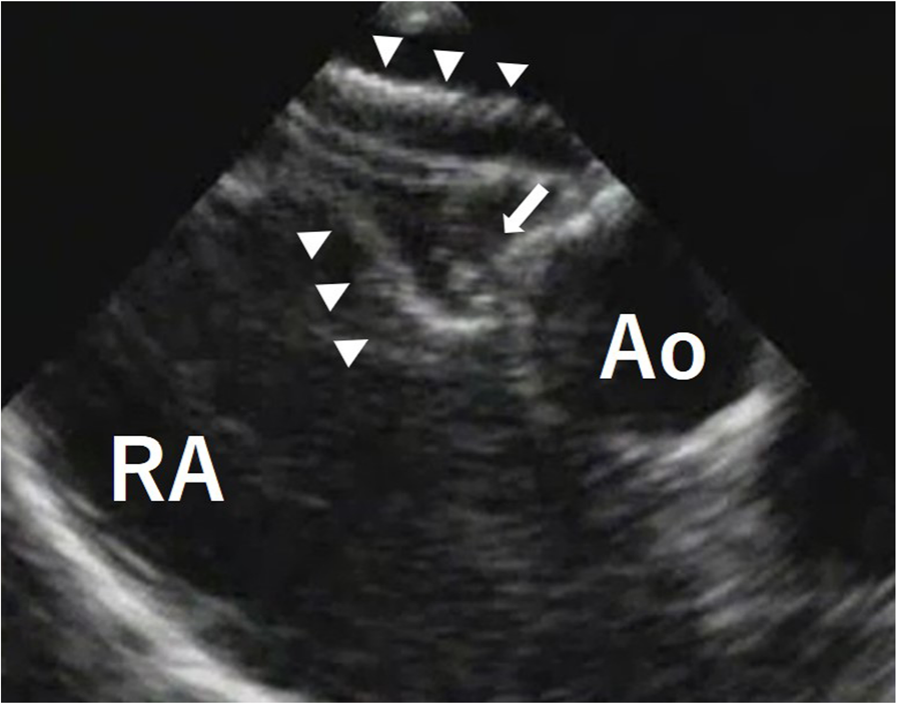
The transesophageal echocardiogram after ASO implantation revealed the “A-shape” of the device (white arrows) and the absence of an aortic rim

**Fig. 2:**
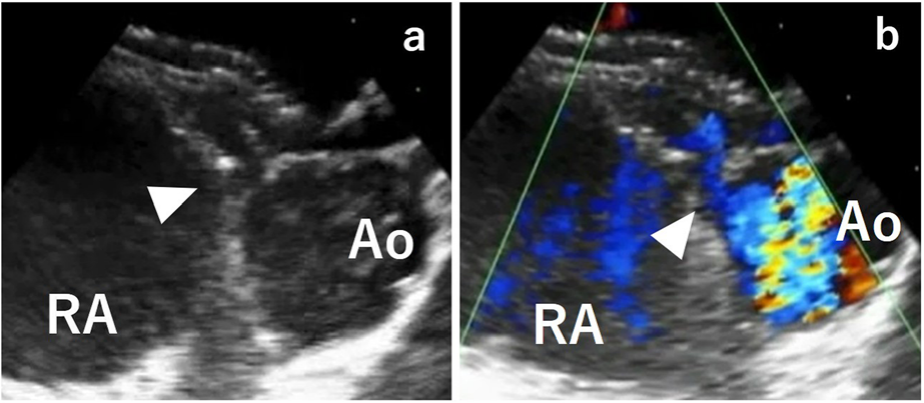
**a**, **b**. The transesophageal echocardiogram during the emergency operation for cardiac tamponade. The edge of the Amplazter septal occluder was touching the aortic wall (**a**. white arrowhead) and Doppler color-flow imaging showed blood flow through this lesion of the aorta (**b**. white arrowhead)

**Fig. 3:**
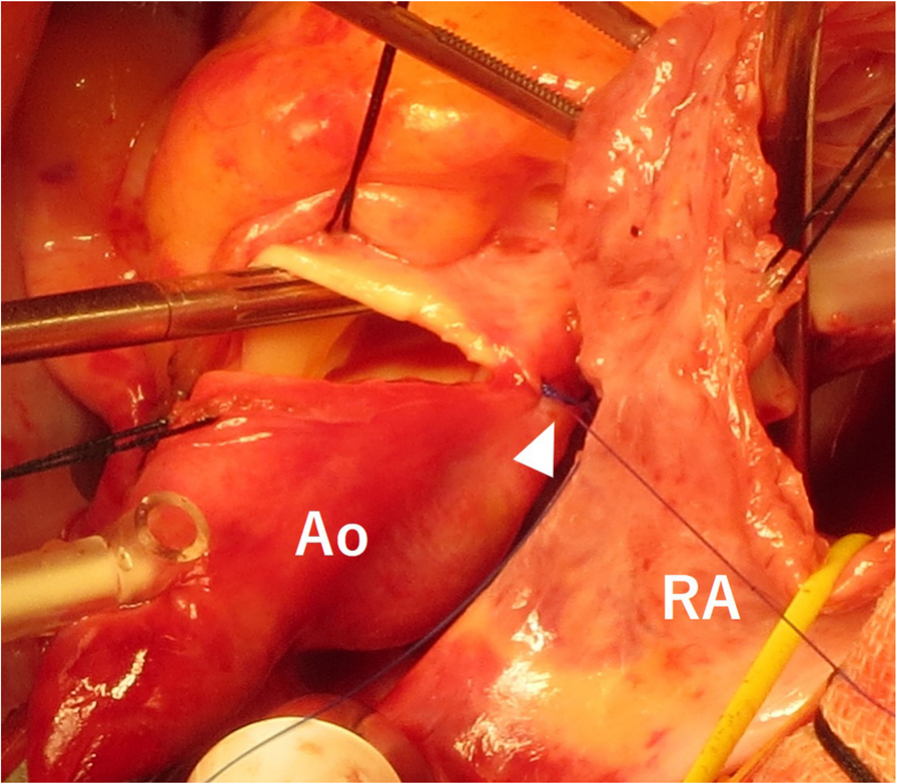
Operative views. A laceration of the aortic wall facing the right atrium was detected and directly sutured (white arrowhead)

**Fig. 4:**
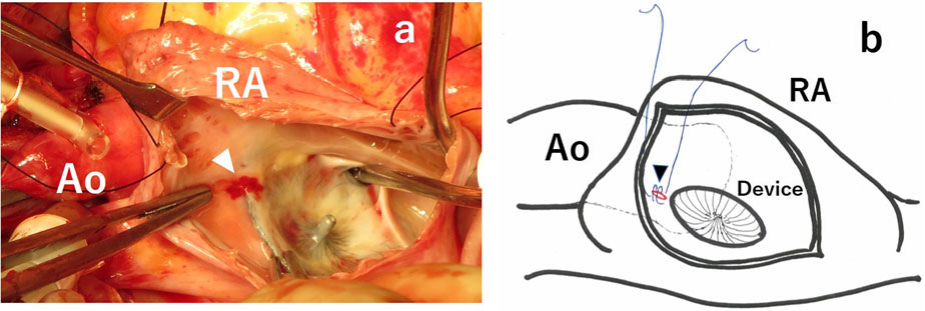
**a**, **b**. Operative views. A laceration induced by Amplazter septal occluder was detected from the inside of the right atrium (**a**. white arrowhead). The scheme shows that the laceration was closed with 5–0 polypropylene continuous sutures (**b**. black arrowhead)

A seven-year-old girl was diagnosed with secundum ASD without symptoms. Transesophageal echocardiography (TEE) showed a defect of 20 mm in diameter in the fossa ovalis without aortic rim. An ASO device of 24 mm in diameter was selected and electively implanted. The “A-shape” of the device was confirmed by intraoperative TEE, a landmark finding indicationg the proper implantation of ASO in patients without aortic rim (Fig. 1). After an uneventful postoperative course of 5 years and 10 months, she was transferred to our unit due to cardiogenic shock. Her echocardiogram in emergency room showed pericardial effusion with a collapsed right ventricle. Given her history of ASO and the observation of sequentially increasing pericardial effusion, we diagnosed her with acute cardiac tamponade due to aortic erosion. Emergency pericardiotomy was then performed to improve the hemodynamic condition. Fresh clots were found, so we immediately prepared the cardiopulmonary bypass circuit and then explored the damage of the aorta, in which the clots were accumulated. Bleeding suddenly started when the clots were removed. We then inserted the cannulae for perfusion and venous drainage. The clots were removed, and tears were found in both the lateral side of the ascending aorta and right atrial wall. Intraoperative TEE showed that an edge of ASO device was directly touching the aortic wall (Fig. 2a), and Doppler color-flow imaging showed blood flow through this lesion (Fig. 2b). The erosive lacerations of both the ascending aorta (Fig. 3) and right atrium (Fig. 4a) were detected from the inside after achieving cardioplegic cardiac arrest. The ascending aorta was obliquely incised, and both lacerations were closed from the inside of the aortic root and right atrium with 5–0 polypropylene continuous sutures (Fig.4b). We removed the ASO device and performed ASD patch closure. The postoperative course was uneventful. She has been doing well for the 5 years since the surgery.

## Discussion and conclusions

ASO implantation is less invasive than the surgical procedure with low morbidity and mortality rates and is thus widely indicated in the treatment of the ASD [1, 2]. However, a major complication with ASO is aortic erosion, which can lead to lethal bleeding. The incidence of this dangerous complication has been reported to be range from 0.1 to 0.3% [1–3], which is not very frequent. However, the mortality rate was reported to be as high as roughly 20% [3].

Regarding the mechanism underlying aortic erosion, Amin et al. strongly speculated the absence of the aortic rim of the defect as being involved. They reported that 25 of 28 patients (89%) with erosion had an aortic rim of < 5 mm [4]. A device that oversized and straddled the aortic root was thus believed to carry a risk of causing erosion. When a patient has a deficient aortic rim, the device tends to be placed by straddling it over the aortic root in order to avoid dislodging. Based on this pathogenesis, the fact that the aortic rim of ASD was deficient and the ASD diameter was 20 mm in our present case suggested a risk of erosion. Given the above, we believe that the ASD in this case should have been surgically treated.

Aortic erosion is most likely to occur 48 to 72 h after device implantation and rarely in the late phase [3–7]. McElhinney et al. also noted that 1/3 of aortic erosion cases developed it within 24 h of implantation, and only 6% developed it over 5 years after implantation [1]. Based on the present and previous findings, we should keep in mind that aortic erosion can occur even years after ASO implantation, and the indication of ASO in cases without a proper aortic rim should be carefully discussed, as ASO implantation is meant to be at least as safe as surgical intervention for ASD.

## Data Availability

The clinical dataset used in this case report are available from the corresponding author on reasonable request.

## Abbreviations

ASD: Atrial septal defect
ASO: Amplazter Septal Occluder
TEE: Transesophageal echocardiography
